# Health and House-Hunting

**Published:** 1874-03

**Authors:** 


### HEALTH AND HOUSE-HUNTING.

Many will select a house this month, for a residence, and it will be their last home on earth; it would not have been, had they remained where they are, or moved elsewhere. It does not express the whole truth to say, that some houses are unhealthy; it is nearer the fact in reference to many dwellings, that they are deadly. Sometimes certain rooms in a house are so impregnated with poisonous emanations, that their occupants become ill in a few days. I know of a capacious mansion (formerly, now a boarding-house) in Walnut-street, Philadelphia, which has in it a certain room, known to make the parties sick within a few days after they move into it. Within a year, a man in perfect health, was placed in a room in London, and in a few days died of putrid fever. The next, and the next, and the next occupant, were noticed successively to become ill. It became so notorious, that the authorities took it in hand to examine the premises, and it was found that the man who papered the room, in order to fill up a cavity in the wall, put in a bucket full of paste and pieces of the glazed papering, which in time began to ferment and rot, throwing into the room a steady supply of the noxious fumes of decomposed lead, and other hurtful ingredients employed in the sizing of wall paper. It is known that the sizing on a visiting card is enough to poison a child if put in its mouth; being a little sweetish to the taste, it is rather palatable.

Another English house became so notoriously unhealthy, that the common people reported it to be haunted; it soon gained such a reputation, that no body would live in it free of rent.

Investigation discovered that it was the result of pasting new paper on old.

Lesson.—*In repapering a room or house, first pull off the old paper, and scrape and wash the walls.*

The Grand Jury of the chief criminal court of New York City used to repeat their bitter complaints against the damp and noisome apartment in which they were compelled to sit day after day in the performance of their official duties. The death of one of their number was attributed by that body to the unhealthfulness of the room they occupied.

The White House at Washington, is believed by observant men there, to be the main reason for the ill-health of our Presidents, since General Harrison first went there, so soon to make it his grave. Its unhealthiness is very justly attributed to the construction of a bridge or causeway across the stream, which passes near it, thus giving a larger body of still water than in former times; and the neighborhood of stagnant water, with the usual amount of decaying vegetation, must originate disease in the warmer portions of the year in all temperate latitudes.

These things being true in reference to houses, there are other items to be taken into consideration in selecting our dwellings, besides price, appearance and neighborhood.

Very many persons in cities are decided, in determining upon a residence for themselves and families, by the appearance of the street front. An elegant frontage of brown stone, towering in stateliness to five stories, brings many a dollar beyond its value to pursy landlords. But how vigorously fond new husbands and weak old ones have to *shin around* in the slops and snows of winter to pay the rent, and "*monstrous*" hard as it may be in winter, summer heats make it "monstrouser," as Charcoal Sketches would say. How many a restless turn at night, how many a Sunday plan, which matter of fact Monday morning makes vanish in thin air, how many an anxious conjecture it costs, whether this acquaintance or that old friend, or nearest neighbor might not make a loan "*on call*," to help out at quarter day; how many racks of self respect, of personal independence, of wounded pride, of debasing tergiversation it costs to pay for this purchase of appearances, the initiated can better tell than I can guess, never having been a renter "in the whole course of my life," except for a short year on trial, in the country; yes, in the country! delightful summer residence! on the banks of the Hudson! just over against the Palisades! as dear a purchase of imaginary blisses, as of the *appearances* aforesaid. I like no half ways, give me the centre of the largest city on the continent, or a log cabin in the far recesses of the unpenetrated west.

But the waste of money to keep up appearances, is not the greatest loss; health sacrificed, life perilled, is often times an "extra" not calculated on, but like "extras," comes with a thunder clap of unexpectedness, meeting too, the fate of all "extras," an exclamation, a demur, dwindling down to an argument and final delivery of the purse strings.

Lesson 2nd.—*Reader, pay extras and be done with it. I have always found it the quickest and the easiest plan.* It saves temper, for the more you argue about it, the more angry you will get, and the worse you will feel afterwards when you find you have not only lost your temper, but your money too.

Other persons, as intimated already, will put jewelry, plate, gold watch, all "up the spout" to make up the usual advance on the first quarter, to the landlord, who has not the pleasure of their acquaintance; will do all this, to secure a residence in a "*genteel street*," or "fashionable neighborhood" on "*the*" side of Broadway. There are men and women, that is, grown persons of both sexes in New York, who would think themselves hopelessly disgraced to live in a street which had "*East*" attached to it; would consider they had lost cast more irrecoverably by living on the "*other*" side of Broadway, than if they had, in a pinch, checked on a bank for ten thousand, when they had never deposited a dollar there. To such persons, and to all others living in cities, I wish to make some suggestions in reference to the selection of a family residence.

If practicable, let the rear of the house face the south; mainly for two reasons, first and chief, unsightly things, the washings of the kitchen and the laundry are deposited there, and with other causes, keep the back yards almost always in a damp condition; which, with the dust and unavoidable accretions of various kinds, make fit materials for decompositions, and their inevitable result, the generation of hurtful gases, sometimes actually poisonous. The heat of the sun has a drying influence, and with moderate attention, the premises may be kept sweet and clean. The second reason is, greater light is afforded to the kitchen, where it is so much needed, especially in winter time, to allow of the cleanly preparation of daily food. A mind of any refinement, revolts at the mere mention of cookery in the dark.

The front of a house in the city does not so much need the sun, since the too frequent custom, is to make a parlor of the first floor front, for the occasional accommodation or reception of guests and visitors, in many instances averaging not an hour a day; and for similar reasons, the "spare rooms," are those in front in the upper stories. In my opinion, the very best, largest and most commodious rooms in a house should be appropriated to the daily and hourly use of the family.

As accumulations are not allowed in the streets, the sun is not so much needed on a northern front, while the passing of persons and vehicles, compensate in cheeriness for the absence of sunshine; but it is not a total absence, for there is the sunshine of the countenance of your visitors; unless of that not innumerous class, who are rather disagreeably disappointed, when they find you are at home, and had much rather have left a card; their smiles are of the sardonic order, or of the mechanical kind, icicling in a moment, all the outgushings of kindliness, were it not the fashion to keep our parlors so dim and dusky, that we can't tell whether the smile comes from the head or the heart.

In selecting a residence, notice if there is any standing water in the cellar, any uncovered drain or well; I know of two adjoining houses in Philadelphia, which have brought death to every family that has occupied them for some years past, and another not far distant which has proved the death of three successive occupants, each of them strong hearty men when they moved in.

Notice the rear premises; if they adjoin a stone cutter, or livery stable, or distillery, or cow yard, or for drays, carriages and the like; if any of these are within a block of you in any direction, the house is dear at any price, it is dear at nothing, whatever may be its frontage.

As a general rule avoid long rows of brown stone fronts, built uniformly; or of brick or any other material; they were built by contract, or for purposes of speculation. If the flues do not burn you up, there is large probability that the rats will devour every thing you purchase, over and above what you actually consume, and the friends whom Biddy your cook supplies with their daily provender. Sometime since I accompanied a gentleman, who wanted to purchase or lease a family mansion, on a tour of observation. We looked through one of a row of five story brown fronts, one of the most imposing in appearance outside in New York; it had been occupied but a year, the flue had set it on fire; the family had left, and there being no carpeting or other furniture to cover defects, there was revealed to us a quality of carpentership utterly disgraceful to both builders and owners; the flooring had not the roughness planed off in many places; while the spaces between the "tongue and grooves," as also between the ends of the planks, and between the wash or surboard and the floor, were in many instances from a quarter to half an inch or more in width; and this, in rooms where the fire and water had no access; these items, together with spoiled locks, broken keys, doors hanging awry from a shrinking of the wood and "settling" of the building, immovable window sash, made a tenement which notwithstanding its fine brown stone frontage, was unfit to be occupied by any family who wanted to live comfortably.

